# From wisdom to efficacy: the mediating role of positivity ratio in the relationship between university students’ knowledge strengths and academic self-efficacy

**DOI:** 10.3389/fpsyg.2025.1705454

**Published:** 2025-11-25

**Authors:** Sitong Li, Mingchun Luo

**Affiliations:** 1 Carey Business School, Johns Hopkins University, Washington, DC, United States; 2 Department of Psychology, School of Education, Yunnan Minzu University, Kunming, Yunnan, China

**Keywords:** character strengths, wisdom and knowledge, positivity ratio, academic self-efficacy, university students

## Abstract

**Background:**

Grounded in positive psychology and the broaden-and-build theory, this study examined how VIA-IS Wisdom and Knowledge predicts academic self-efficacy via the positivity ratio, distinguishing Learning Ability Efficacy and Learning Behavior Efficacy. Drawing on positive psychology and the broaden-and-build theory, this study tested a mechanism model linking *wisdom and knowledge strengths → positivity ratio → academic self-efficacy*. Two facets of academic self-efficacy were distinguished—learning ability efficacy and learning behavior efficacy—to clarify whether emotions contribute differently to confidence in understanding versus managing learning tasks.

**Methods:**

A cross-sectional online survey was conducted with 531 Chinese university students (73% female, 27% male; 34% urban Hukou, 66% rural; recruited from five comprehensive/normal universities in southwestern China across education, psychology, management, and engineering majors). Measures included the wisdom and knowledge dimension of the VIA-IS, the revised Chinese Positive and Negative Affect Schedule for computing positivity ratio, and the Academic Self-Efficacy Scale. Data were analyzed by SPSS 27.0 using correlation, mediation and multivariate GLM analyses to test the hypothesized strengths → emotions → efficacy pathway.

**Results:**

Students with higher wisdom and knowledge strengths and higher positivity ratios reported stronger academic self-efficacy (*r* = 0.42–0.47, *p* < 0.01). Emotional balance partially mediated this link [indirect effect = −0.06, 95% CI (−0.11, −0.03)], suggesting that positive affect serves as a bridge between cognitive strengths and confidence in learning. This emotional pathway was significantly stronger for ability-related efficacy than for behavior-related efficacy (Δ = 0.10, *p* < 0 0.001), indicating that feeling positive is especially crucial for beliefs about learning competence.

**Conclusion:**

The correlation between wisdom and knowledge strengths and academic self-efficacy is twofold: direct and indirect. The direct correlation is evident through positive emotions, while the indirect correlation manifests through the enhancement of academic self-efficacy. The findings emphasize that cognitive strengths and emotional balance collectively foster students’ motivation and learning confidence. Interventions that integrate strength development, emotional regulation, and self-efficacy building may therefore be particularly effective in higher education settings.

## 1 Introduction

As higher education becomes increasingly universalized, university students face growing pressures related to academic evaluation, career competition, and social adaptation. Academic stress is strongly associated with adverse outcomes such as anxiety, depression, and burnout, and it can reduce learning engagement and performance (Bayram and Bilgel, 2008; Beiter et al., 2015; Pascoe et al., 2020). How to maintain motivation and mental health under such high-pressure conditions has become a central concern in educational psychology and student support services (Zimmerman, 2000; Schunk and DiBenedetto, 2020). Recent epidemiological data underscore the urgency: a 2025 BMC Public Health survey of over 49,000 Chinese undergraduates found prevalence rates of 9.8% for depressive symptoms and 15.5% for anxiety (Han et al., 2025). This national-level context amplifies the need for psychological resources that can buffer pressures in academic settings. Recent research suggests that the pursuit of happiness may not uniformly enhance wellbeing, as its outcomes depend on underlying goal orientations and contextual factors (Yıldırım et al., 2022).

This study focuses on three key constructs. First, academic self-efficacy (ASE) refers to students’ beliefs in their ability to complete academic tasks. ASE predicts learning choices, effort, persistence, and achievement (Bandura, 1997; Zimmerman, 2000), and its effect on performance has been confirmed in meta-analyses (Honicke and Broadbent, 2016). Meta-analytic evidence confirms its consistent predictive power across contexts (Honicke and Broadbent, 2016). Two distinct facets are delineated: learning ability efficacy (Y₁) denotes confidence in mastering academic content, and learning behavior efficacy (Y₂) denotes confidence in planning, time management, and regulation (Pekrun et al., 2002; Pintrich, 2000). These conceptual splits parallel contemporary models that separate competence beliefs from self-regulatory enactment, allowing more precise mapping between beliefs and proximal learning processes (Schunk and DiBenedetto, 2020). Recent evidence highlights the centrality of ASE as a psychological mediator linking social support, resilience, and academic adjustment (Green et al., 2024a). Second, wisdom and knowledge strengths, drawn from the Values in Action (VIA) framework, encompass creativity, curiosity, critical thinking, love of learning, and perspective (Peterson and Seligman, 2004; Park and Peterson, 2006). These strengths are positively related to classroom engagement, learning enjoyment, ASE, and achievement (Wagner and Ruch, 2015; Lavy, 2020). The efficacy of experimental programs designed to cultivate creativity and intellectual strengths has demonstrated marked gains in students’ self-efficacy and academic motivation (Green et al., 2024b). Emerging intervention research indicates that strength-based programming can improve wellbeing, post-traumatic growth, and psychological adjustment among university students (Green et al., 2022; Yu et al., 2022). Concurrently, self-efficacy has been demonstrated to mitigate the emotional consequences of fear and uncertainty, thereby functioning as a pivotal protective factor for students’ mental wellbeing (Green et al., 2024c). Third, the positivity ratio, defined as the ratio of positive to negative affect (PA/NA), reflects emotional balance. The Positive and Negative Affect Schedule (PANAS) has shown good validity in Chinese student samples (Guo and Gan, 2010), and its reliability has also been confirmed in large non-clinical samples (Crawford and Henry, 2004). Emotional balance is linked with wellbeing, resilience, and motivation (Fredrickson and Joiner, 2002). In academic contexts, positive affect facilitates exploration and flexible strategy use, whereas negative affect is associated with narrowed attention and avoidance tendencies (Isen, 1993). Extant research lends further support to this affective mechanism: prosocial tendencies and hope enhance emotional autonomy and wellbeing through serial mediation processes (Eryılmaz et al., 2025), while resilience and social support jointly promote flourishing among youth (Yıldırım and Green, 2024). Recent work in educational settings shows that affective indices assessed with PANAS-type measures are linked to motivation and engagement outcomes—for example, positive affect has been found to mediate the effect of trait emotional intelligence on academic engagement (Sanchez-Ruiz et al., 2024) and to function as a key pathway from proactive personality to online learning engagement (Fu et al., 2024). Cross-cultural validation studies also support the generalizability of wellbeing mechanisms across diverse educational and cultural contexts (Jovanović et al., 2024).

The interconnections among these constructs are grounded in established theories. Social cognitive theory emphasizes emotional states as a critical source of efficacy beliefs, shaping how individuals interpret their abilities, persistence, and control in learning contexts (Bandura, 1997; Pekrun et al., 2002). The broaden-and-build theory posits that positive emotions broaden attention, cognitive flexibility, and working memory, thereby creating an “upward spiral” of resource accumulation (Isen, 1993; Fredrickson, 1998; Fredrickson and Joiner, 2002). The conservation of resources theory similarly highlights gain spirals, suggesting that strengths such as wisdom and knowledge can enhance mastery directly and indirectly via positive emotions (Hobfoll, 2001). Taken together, these accounts imply a coherent pathway—strengths cultivate cognitive–motivational resources, emotions broaden cognitive scope, and efficacy beliefs consolidate adaptive engagement—yet empirical tests that integrate all three levels in one model remain scarce. Furthermore, studies in various cultural and adversity contexts highlight the central role of personal and social resources in promoting psychological growth and adjustment. For example, self-reported data from children in Pakistani care homes indicates that supportive relationships and self-reflection can promote post-traumatic growth even under severe adversity (Masoom Ali et al., 2020). Similarly, cross-cultural research in Turkey emphasizes the significance of adaptive adjustment processes in maintaining mental health (Yıldırım and Solmaz, 2020). Additionally, studies with Saudi and Middle Eastern participants reveal that gratitude and self-esteem are key contributors to subjective wellbeing (Yildirim et al., 2019). Together, these findings emphasize that internal strengths and external support systems dynamically interact to sustain resilience and flourishing across different sociocultural environments.

Despite these links, prior research has limitations. First, many studies have examined single pathways of ASE development, without integrating strengths and emotions into a unified model (Zimmerman, 2000; Schunk and Di Benedetto, 2020). Second, while both strengths and emotions relate to ASE, little research has tested whether emotional balance mediates the effect of strengths on efficacy within the same sample and across distinct ASE dimensions (Quinlan et al., 2012; Wagner and Ruch, 2015; Lavy, 2020). Third, potential differential mediation remains unexplored: positive emotions may more directly enhance ability-oriented efficacy (Y₁) through immediate cognitive benefits, while behavioral efficacy (Y₂) may depend more on self-regulatory strategies, influenced indirectly by affect (Ashby and Isen, 1999; Pintrich, 2000). Moreover, most available studies are cross-sectional and treat ASE as a unidimensional construct, which can mask pathway heterogeneity and inflate or deflate indirect effects (Honicke and Broadbent, 2016).

Accordingly, this study proposes a mechanism model: strengths → emotions → efficacy—that integrates wisdom and knowledge strengths, positivity ratio, and two ASE dimensions. The following hypothesis is postulated: H1, wisdom and knowledge strengths, and positivity ratio will be positively associated with both Y₁ and Y₂ (Zimmerman, 2000; Fredrickson and Joiner, 2002; Wagner and Ruch, 2015). H2, positivity ratio will mediate the relationship between wisdom and knowledge strengths and ASE (Bandura, 1997; Fredrickson, 2001; Pekrun et al., 2002). H3, the mediating effect will be stronger for Y₁ than for Y₂, consistent with the immediate cognitive benefits of positive affect (Ashby and Isen, 1999; Pintrich, 2000). By distinguishing efficacy facets and explicitly modeling emotion as a mediator, the present work aims to move beyond bivariate associations and provide a more mechanistic account of how character strengths translate into adaptive beliefs in real academic settings.

**Figure 1:**
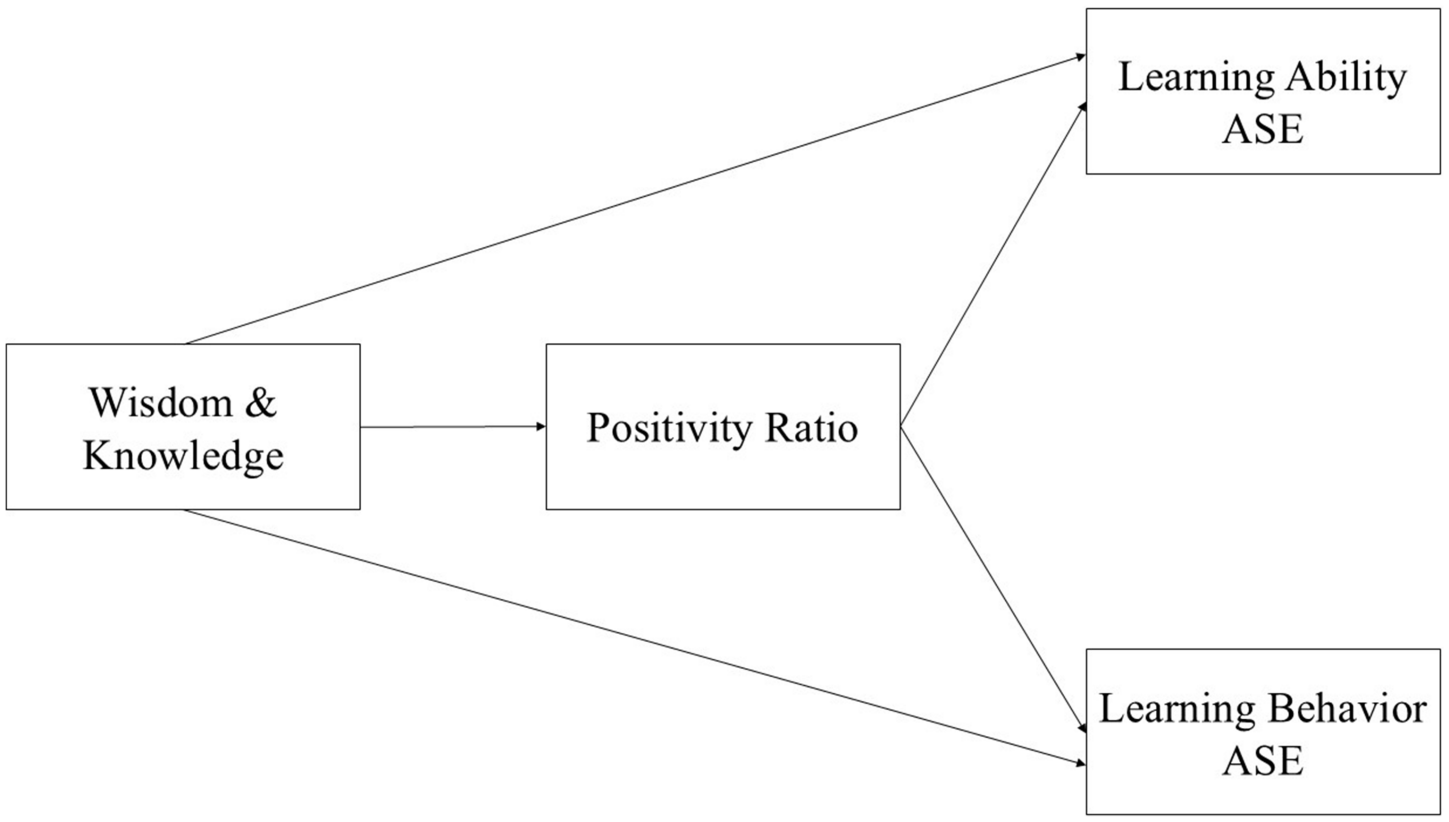
Hypothesized model: wisdom and knowledge strengths → positivity ratio → ASE.

The contribution of this study lies in clarifying how strengths, emotions, and efficacy interact within a unified framework. By testing both mediation and differential effects, this research provides integrated evidence for educational practice, highlighting the potential of combining strengths cultivation, emotional interventions, and belief construction to support students’ academic development. The hypothesized model is presented in Figure 1. In practical terms, such an integrated approach aligns with scalable school-based programs that target strengths identification, emotion regulation, and strategy coaching in tandem (Quinlan et al., 2012; Lavy, 2020).

## 2 Materials and methods

### 2.1 Present study

Although prior studies have highlighted the significance of positive emotions and character strengths in fostering students’ academic success and wellbeing, there is a paucity of research that has examined how these factors collectively influence academic self-efficacy, a pivotal predictor of learning motivation and achievement. The broaden-and-build theory (Fredrickson, 2001) posits that positive emotions augment cognitive and motivational resources. In contrast, social cognitive theory (Bandura, 1997) underscores emotional states as pivotal sources of efficacy beliefs. The present study integrates these perspectives and focuses on the wisdom and knowledge dimension of the VIA-IS. This dimension represents cognitive strengths such as curiosity, creativity, and critical thinking. These strengths may cultivate adaptive learning confidence through emotional balance.

Despite the established correlation between positive emotions and academic engagement in previous research, the majority of studies have approached ASE as a unidimensional construct, seldom differentiating between learning ability efficacy (i.e., confidence in content mastery) and learning behavior efficacy (i.e., confidence in time management and effort). Furthermore, the utilization of the positivity ratio (PA/NA) as an indicator of affective balance remains a subject of debate due to its mathematical sensitivity, necessitating additional empirical validation through the implementation of complementary measures.

The objective of this study is to: Firstly, it is necessary to elucidate the manner in which wisdom and knowledge strengths influence ASE through affective balance. Secondly, it is imperative to examine whether the mediating effect of positive affect differs between learning ability and behavior efficacy. Thirdly, the robustness of these relationships must be tested using both ratio- and difference-based affect indicators. In accordance with these objectives, the following three hypotheses were proposed:

*H1*: Wisdom and knowledge strengths are positively associated with both learning ability and learning behavior efficacy.

*H2*: The positivity ratio mediates the relationship between wisdom and knowledge strengths and ASE.

*H3*: The indirect effect of positive affect is stronger for learning ability efficacy than for learning behavior efficacy.

### 2.2 Participants

This study adopted a cross-sectional design using convenience sampling via an online survey. The questionnaire was distributed through Wenjuanxing (So Jump), a widely used online survey platform in China, to recruit full-time undergraduates, master’s, and doctoral students from five comprehensive and normal universities located in a southwestern border province of China, covering majors in education, psychology, management, and engineering (July to September 2025).

According to predefined inclusion and exclusion criteria—(i) incomplete responses, (ii) response time less than 50% of the sample median, and (iii) straight lining within any scale—57 responses were excluded, yielding a final valid sample of 531 out of 588 participants. The sample consisted of 145 males (27.31%) and 386 females (72.69%). Regarding household registration, 179 students (33.71%) reported an urban hukou and 352 students (66.29%) reported a rural hukou. The gender imbalance reflects the overrepresentation of female students in education- and psychology-related majors in Chinese universities and should be considered when interpreting generalizability. Preliminary *t*-tests analyses revealed no significant mean differences in key study variables between gender and hukou groups (*p*s > 0.05, *d*s < 0.40). These results are shown at Table 1, which suggest that the main variables remained consistent across demographic subgroups, which supports the generalizability of subsequent analyses. Table 1 presents the descriptive statistics and group comparisons by gender and hukou status. Convenience sampling may also limit representativeness, though the sample size was sufficient for mediation testing. *A priori* sensitivity checks indicated that with *N* = 531 and *α* = 0.05, the study had >0.95 power to detect small-to-medium indirect effects in simple mediation (Fritz and MacKinnon, 2007).

**Table 1:** Descriptive statistics and group comparisons across gender and hukou (*n* = 531).

Variable	Male	Female	*t*-value	*d*	Urban	Rural	*t*-value	*d*
					*M* (*SD*)	*M* (*SD*)		
	*M* (*SD*)	*M* (*SD*)						
Positive affect	3.08 (0.93)	3.05(0.83)	0.47	0.05	3.15 (0.96)	3.01 (0.80)	1.88	0.17
Negative affect	2.11 (0.86)	2.30 (0.91)	−2.12	0.21	2.03 (0.93)	2.35 (0.86)	−3.95^***^	0.36
Positivity ratio	1.70 (0.90)	1.55 (0.83)	1.86	0.18	1.87 (1.04)	1.45 (0.70)	5.54^***^	0.51
Learning ability efficacy	3.69 (0.81)	3.48 (0.77)	2.81^***^	0.27	3.73 (0.78)	3.44 (0.77)	4.20^***^	0.39
Learning behavior efficacy	3.40 (0.58)	3.33 (0.49)	1.37	0.13	3.52 (0.61)	3.26 (0.44)	5.65^***^	0.52
Wisdom and knowledge	2.91 (0.58)	2.87 (0.60)	0.60	0.06	2.89 (0.59)	2.88 (0.59)	0.27	0.03

Independent-samples *t*-tests were conducted to compare gender and hukou differences; ^***^Correlation is significant at the 0.001 level. ^**^Correlation is significant at the 0.01 level (2-tailed). “d” means Cohen’s *d* value.

Survey quality was ensured by Wenjuanxing functions that recorded response times and timestamps. After reading and agreeing to the informed consent information, all core instruments were set as mandatory and presented in a fixed order: VIA-IS Wisdom and Knowledge dimension → PANAS (Chinese revised version, for positivity ratio = PA/NA) → TSES (Learning Ability ASE and Learning Behavior ASE) → demographics. Data were exported and screened for straight lining and abnormal durations before analysis. To mitigate common-method concerns, instructions emphasized honest responding and anonymity, item anchors were varied where appropriate, and analyses later included a single-factor test as a diagnostic (Podsakoff et al., 2003).

### 2.3 Measures

In the present study, the internal consistency coefficients (Cronbach’s *α*) of all measures are reported in the Results section. The complete item lists of the Academic Self-Efficacy Scale (TSES) (Liang, 2000) and the revised Positive and Negative Affect Schedule (PANAS) (Guo and Gan, 2010) are provided in Supplementary material. Descriptive wording adjustments were made in accordance with standard guidelines to maintain functional equivalence while preserving the original construct definitions. All instruments have been previously validated among Chinese university students, ensuring cultural and linguistic appropriateness for the present context.

#### 2.3.1 Character strengths—wisdom and knowledge dimension

Character strengths were measured using the Chinese wording of the Values in Action Inventory of Strengths (VIA-IS) (Peterson and Seligman, 2004), focusing on the Wisdom and Knowledge virtue. Within the VIA framework, this virtue comprises five cognitive–inquisitive strengths: Creativity, Curiosity, Judgment/Critical thinking, Love of Learning, and Perspective. An example item is “When others tell me how to do something, I unconsciously think of other ways.” Each strength is represented by items in the full VIA-IS, rated on a 5-point Likert scale (1 = “very much unlike me” to 5 = “very much like me”). The Cronbach’s *α* for this study was 0.80. Cross-cultural studies have demonstrated that the VIA-IS possesses adequate to good psychometric properties across languages and populations (Shryack et al., 2010; Littman-Ovadia and Lavy, 2012; McGrath, 2015).

For application in Mainland China, a validated Chinese wording of the VIA-IS was used, which has been tested among Chinese university students and shown acceptable structural validity and internal consistency (Duan et al., 2011). Additional evidence has supported the psychometric adequacy of Chinese VIA measures in youth and educational contexts (Cheng et al., 2022). In the present study, a composite score for Wisdom and Knowledge was calculated by averaging item scores across the five strengths, with higher scores indicating stronger endorsement of this virtue. Item parcels were not used; treating the virtue as a composite avoids over-parameterization relative to sample size in subsequent models.

#### 2.3.2 Positivity ratio

The positivity ratio was derived from the revised Chinese version of the Positive and Negative Affect Schedule (PANAS; Guo and Gan, 2010). The scale consists of 18 items, divided into 9 for positive affect (e.g., “Active”) and 9 for negative affect (e.g., “Ashamed”), each rated on a 5-point Likert scale (1 = very slightly or not at all, 5 = extremely). Prior research has demonstrated good reliability and construct validity in Chinese samples (Guo and Gan, 2010) and in Western populations (Crawford and Henry, 2004). Cronbach’s *α* = 0.95 for PA and 0.95 for NA in this sample. The positivity ratio was computed as:


$$\text{Positivity Ratio}=\frac{\text{Positive Affect}\;(\mathrm{PA})}{\text{Negative Affect}\;(\mathrm{NA})}$$

Higher values indicate a greater proportion of positive affect. The positivity ratio was treated as a continuous index without applying any cutoff threshold. In light of the ongoing discourse surrounding the mathematical sensitivity of ratio-based affect indices (Brown et al., 2013), a robustness check was incorporated into the positivity ratio using the difference score (PA–NA). The utilization of both indices in the representation of affective balance has been extensive, and the consistent patterns they exhibit in subsequent analyses lend support to the reliability of the emotional measurement approach.

#### 2.3.3 Academic self-efficacy

Academic self-efficacy was measured with the Academic Self-Efficacy Scale (TSES) (Liang, 2000). The instrument consists of 22 items rated on a 5-point Likert scale (1 = strongly disagree, 5 = strongly agree). It includes two subscales: Learning Ability ASE (e.g., “I believe I can achieve high academic performance”), reflecting judgments of capability to master academic content, and Learning Behavior ASE (e.g., “I test my knowledge by self-questioning during study sessions”), reflecting confidence in planning, time management, and behavioral regulation. The Cronbach’s α was 0.72 for learning ability and 0.95 for learning behavior subscales.

Studies conducted in Chinese university student samples have consistently reported satisfactory internal consistency (typically *α* ≥ 0.80) and structural validity for the TSES (Liang, 2000; Zhen et al., 2017). Higher scores indicate stronger perceived academic self-efficacy. Subscale scores were analyzed separately to preserve facet specificity central to the hypotheses.

### 2.4 Procedure

Data collection was carried out through Wenjuanxing, an online survey platform widely used in China. The questionnaire was accessible on both mobile and desktop devices. To enhance representativeness and heterogeneity, participants were recruited from multiple universities representing different disciplines (social sciences, education, and management) in southwestern China. Prior to the formal survey, a small-scale pilot (100 participants) was conducted to check the clarity and comprehensibility of the items, and minor adjustments were made accordingly. Attention-check prompts were embedded (e.g., “Please select “agree” for this item”) to flag inattentive responding; flagged cases were removed under the straight lining/quality rules.

In the primary survey, items were presented in a fixed sequence: (1) VIA-IS Wisdom and Knowledge dimension, (2) revised Chinese PANAS, (3) TSES Academic Self-Efficacy Scale, and (4) demographic questions (gender, year of study, hukou type). The average completion time was approximately 5 min. The survey was open from July to September 2025. Participation was voluntary, with informed consent obtained electronically before respondents proceeded. No identifiable personal information was collected, and responses were treated anonymously and confidentially. The study procedures complied with the ethical guidelines of the American Psychological Association (Code of Psychologists, 2017). Institutional review procedures followed local norms for minimal-risk online surveys with adult participants.

### 2.5 Data analysis

All statistical analyses were conducted using IBM SPSS Statistics 27.0 and the PROCESS Macro v4.1 (Hayes, 2013).

First, data screening and descriptive analyses were performed, including means (M), standard deviations (SD), and distributional characteristics of all variables. Internal consistency reliability (Cronbach’s *α*) is reported in the Results section (Nunnally and Bernstein, 1994). Assumption checks included inspection of outliers, normality (skewness/kurtosis and Q–Q plots), and multicollinearity diagnostics (tolerance, VIF < 5) (Field, 2024).

Second, Pearson correlation analyses were used to examine the associations between wisdom and knowledge, positivity ratio, and both dimensions of ASE, corresponding to H1.

Third, mediation analyses (H2) were tested using PROCESS Macro Model 4, with Learning Ability ASE and Learning Behavior ASE entered separately as dependent variables. Bootstrap estimation with 5,000 resamples was applied to generate 95% confidence intervals (CIs). An indirect effect was considered significant if the CI did not include zero. Standardized coefficients (*β*) were reported for total, direct, and indirect effects (Hayes, 2013).

To evaluate robustness, the analyses were repeated with the PA–NA difference score as an alternative affect balance indicator (Brown et al., 2013), yielding comparable results.

Finally, to compare the strength of the effects of the positivity ratio on the two ASE dimensions (H3), a multivariate General Linear Model (GLM) was conducted with both ASE outcomes entered simultaneously. Wisdom and positivity ratio were included as covariates. Custom contrasts (/MMATRIX and /LMATRIX) were specified to test differences between the slopes of positivity ratio predicting Y₁ and Y₂. Point estimates, standard errors, 95% CIs, *F* values, and partial *η^2^* were reported. For comparability, regression coefficients of the positivity ratio predicting each ASE dimension were also provided. All tests were two-tailed, with a significance threshold set at *p* < 0.05. Effect sizes (*r*, *β*, partial *η^2^*) were interpreted in relation to conventional benchmarks (Cohen, 2013).

## 3 Results

### 3.1 Descriptive statistics and reliability

Six continuous variables were analyzed: VIA-IS Wisdom and Knowledge (X), PANAS Positive Affect (PA), PANAS Negative Affect (NA), the Positivity Ratio (*M* = PA/NA), TSES Learning Ability ASE (Y₁), and TSES Learning Behavior ASE (Y₂). Descriptive statistics are presented in Table 2. Mean values were as follows: Wisdom and Knowledge (*M* = 2.88, SD = 0.59), PA (*M* = 2.25, *SD* = 0.90), NA (*M* = 3.06, *SD* = 0.86), Positivity Ratio (*M* = 1.59, *SD* = 0.85), Learning Ability ASE (*M* = 3.35, *SD* = 0.52), and Learning Behavior ASE (*M* = 3.54, *SD* = 0.78). Internal consistency was acceptable to excellent for all multi-item scales: *α* = 0.80 for Wisdom and Knowledge, *α* = 0.95 for PA, *α* = 0.95 for NA, *α* = 0.72 for Learning Ability ASE, and *α* = 0.95 for Learning Behavior ASE. The reliability of the ASE Learning Ability subscale (*α* = 0.72) was comparatively low in relation to other instruments. While this finding remains within the established “acceptable” parameters, it has the potential to compromise the precision of the estimate. The positivity ratio is a computed index (PA/NA) and therefore lacks internal consistency reliability. Skewness values ranged from −0.42 to 0.88, and kurtosis values from −0.31 to 0.95, indicating approximate normality. No univariate outliers were identified, and multicollinearity diagnostics showed tolerance >0.20 and VIF < 5 (Field, 2024). Visual inspection of distributions (skewness, kurtosis, and Q–Q plots) revealed no substantial deviations from normality, supporting the use of Pearson correlations, regression analyses, mediation models, and multivariate GLM.

**Table 2:** Descriptive statistics and internal consistency (*n* = 531).

Variables	Number of items	Minimum	Maximum	*M*	*SD*	Cronbach’s *α*
VIA-IS Wisdom and knowledge (X)	10	1.00	5.00	2.88	0.59	0.80
PANAS—Positive affect (PA)	9	1.00	5.00	2.25	0.90	0.95
PANAS—Negative affect (NA)	9	1.00	5.00	3.06	0.86	0.95
Positivity ratio (PA/NA) (M)	–	0.22	5.00	1.59	0.85	–
TSES—Learning ability ASE (Y₁)	11	1.73	4.82	3.35	0.52	0.72
TSES—Learning behavior ASE (Y₂)	11	1.00	5.00	3.54	0.78	0.95

The positivity ratio is a computed index (PA/NA) and therefore lacks internal consistency reliability.

In addition, according to Cohen (2013) benchmarks, the internal consistency of PA (*α* = 0.95) and NA (*α* = 0.95) was “excellent,” Wisdom and Knowledge (*α* = 0.80) was “good,” Learning Behavior ASE (*α* = 0.95) was “excellent.” Learning Ability ASE (*α* = 0.72) was “acceptable–good.” Visual diagnostics (skewness/kurtosis, Q–Q plots) and outlier screening revealed no substantive threats to later parameter estimates; multicollinearity checks confirmed that all predictors were within safe thresholds (Tolerance >0.20; VIF < 5) (Field, 2024).

### 3.2 Correlation analysis

Pearson correlation coefficients (Table 3) indicated significant associations among cognitive strengths, emotional balance, and academic self-efficacy. The positivity ratio correlated positively with both ASE dimensions: *r* = 0.52 (*p* < 0.001) for Learning Ability ASE (Y₁) and *r* = 0.42 (*p* < 0.001) for Learning Behavior ASE (Y₂). Wisdom and Knowledge strengths were also positively correlated with both ASE dimensions: *r* = 0.38 (*p* < 0.001) with Y₁ and *r* = 0.25 (*p* < 0.001) with Y₂. A modest negative correlation was observed between Wisdom and Knowledge and the Positivity Ratio (*r* = −0.11, *p* = 0.010). The small negative a path is consistent with a competitive (inconsistent) mediation pattern (Zhao et al., 2010): while cognitive–exploratory strengths enhance efficacy directly, they may also covary with higher self-standards/critical appraisal under pressure, temporarily dampening positive affect balance, which partially offsets the direct positive effect—yet the total motivational system remains adaptive. Together, these findings provide initial support for Hypothesis 1, which posits positive associations between wisdom and knowledge strengths, positivity ratio, and both dimensions of ASE.

**Table 3:** Correlations among wisdom and knowledge (X), positivity ratio (M), learning ability ASE (Y₁), and learning behavior ASE (Y₂) (*n* = 531).

	X	M	Y₁	Y₂
X	1			
M	−0.11^**^	1		
Y₁	0.38^***^	0.42^***^	1	
Y₂	0.25^***^	0.52^***^	–	1

^***^Correlation is significant at the 0.001 level; ^**^Correlation is significant at the 0.01 level (2-tailed). X = wisdom and knowledge; M = positivity ratio; Y₁ = learning ability ASE; Y₂ = learning behavior ASE.

In terms of effect size interpretation, *r* = 0.52 (M with Y₂) and *r* = 0.42 (M with Y₁) correspond to “medium-to-large” and “medium” associations, respectively, while correlations of Wisdom and Knowledge with ASE (*r* = 0 0.38; *r* = 0 0.25) correspond to small-to-medium magnitudes under conventional benchmarks (Cohen, 2013). This suggests that both emotional balance and cognitive strengths make practically meaningful contributions to ASE, with emotional balance showing a stronger link to behavior-related efficacy. To reduce concerns of common-method bias, a Harman’s single-factor test was conducted; the first component accounted for substantially less than 50% of the variance (Podsakoff et al., 2003), suggesting that common-source variance did not dominate the observed correlation structure.

### 3.3 Mediation analysis

Mediation models (PROCESS Model 4 with 5,000 bootstrap resamples) tested the indirect role of positivity ratio between Wisdom and Knowledge (X) and ASE dimensions (Y₁, Y₂). The results of the mediation analysis are shown at Table 4. For Y₁, X negatively predicted M (*a* = −0.16, *SE* = 0.06, *p* = 0.010), while M positively predicted Y₁ controlling for X (*b* = 0.43, *SE* = 0.03, *p* < 0.001). The direct effect of X on Y₁ remained significant (*c′* = 0.57, *SE* = 0.05, *p* < 0.001). The indirect effect was significant and negative [*a·b* = −0.07, 95% CI (−0.11, −0.03)], consistent with competitive mediation. For Y₂, X again negatively predicted M (*a* = −0.16, *SE* = 0.06, *p* = 0.010), and M positively predicted Y₂ controlling for X (*b* = 0.33, *SE* = 0.02, *p* < 0.001). The direct effect of X on Y₂ was significant (*c′* = 0.27, *SE* = 0.03, *p* < 0.001). The indirect effect was also significant and negative [*a·b* = −0.05, 95% CI (−0.09, −0.02)], reflecting competitive mediation again. Results therefore supported Hypothesis 2: positivity ratio mediated the relationship between wisdom and knowledge and both ASE dimensions, though in an inconsistent form.

**Table 4:** Mediation analysis (*n* = 531).

Effect	Coeff	Std. Coeff	*SE*	95% CI
X → M → Y₁				
Path a: X → M	−0.16	−0.11	0.06	(−0.28, −0.04)
Path b: M → Y₁ (X)		0.03	0.43	(0.37, 0.49)
Direct c′: X → Y₁ (M)		0.05	0.57	(0.48, 0.66)
Total c: X → Y₁	0.50	0.38	0.05	(0.40, 0.61)
Indirect a·b	−0.07	−0.05	–	(−0.11, −0.03)
X → M → Y₂				
Path a: X → M	−0.16	−0.11	0.06	(−0.28, −0.04)
Path b: M → Y₂ (X)		0.02	0.33	(0.29, 0.37)
Direct c′: X → Y₂ (M)		0.03	0.27	(0.21, 0.33)
Total c: X → Y₂	0.22	0.25	0.04	(0.15, 0.29)
Indirect a·b	−0.05	−0.062	–	(−0.09, −0.02)

X = wisdom and knowledge; M = positivity ratio; Y₁ = learning ability efficacy; Y₂ = learning behavior efficacy.

In terms of effect size, the inconsistent mediation for Y₁ (*a·b* = −0.07, *β* = −0.052) was in the “small-to-medium” range, while the effect for Y₂ (*a·b* = −0.05, *β* = −0.062) also fell within the “small-to-medium” range (Cohen, 2013). To provide standardized effect size benchmarks, the indirect effect for Y₁ corresponded to *β* = −0.052, *k^2^* = 0.032, and *PM* = 0.12, indicating that approximately 12% of the total effect of wisdom and knowledge on ability efficacy was transmitted through affective balance. This indicates that although direct positive effects of Wisdom and Knowledge on ASE remain strong, the emotional pathway partially inhibits the total effect, reducing overall magnitude (Zhao et al., 2010).

In order to circumvent the potential for structural randomness, a robustness check was conducted. In light of the persistent discourse surrounding the mathematical sensitivity of PA/NA ratios to diminutive denominators (Brown et al., 2013), the mediation analyses were re-estimated employing the PA–NA difference score in lieu of the ratio. The pattern of significant paths and confidence intervals remained virtually unchanged, thereby confirming the robustness of the competitive mediation pattern across affect-balance indicators. As a robustness check, the mediation models were re-estimated using the PA–NA difference score instead of the PA/NA ratio. As demonstrated in Table 5, the direction, magnitude, and significance of all paths remained virtually unchanged, indicating that the competitive mediation pattern was robust across affect-balance indicators.

**Table 5:** Robustness check: comparison of ratio vs. difference indicators (*n* = 531).

Outcome variable	Mediator type	*β* Path a	*β* Path b	Direct effect	Indirect effect	*β* indirect	95% CI
Y₁	PA/NA ratio	−0.16^***^	0.43^***^	0.57^***^	−0.07	−0.052	(−0.11, −0.03)
	PA–NA difference	−0.22^**^	0.38^***^	0.58^***^	−0.07	−0.056	(−0.13, −0.02)
Y₂	PA/NA ratio	−0.11^**^	0.33^***^	0.273^***^	−0.05	−0.061	(−0.09, −0.02)
	PA–NA difference	−0.22^**^	0.241^***^	0.27^***^	−0.05	−0.060	(−0.09, −0.02)

^***^Correlation is significant at the 0.001 level; ^**^Correlation is significant at the 0.01 level (2-tailed). Y₁ = learning ability efficacy; Y₂ = learning behavior efficacy; Path a = (wisdom and knowledge→ positivity ratio); Path b = (positivity ratio→ outcome variable).

### 3.4 Comparison of mediation magnitudes

A multivariate GLM tested whether the strength of the positivity ratio’s effect differed between Y₁ and Y₂. Regression slopes indicated a more substantial effect of M on Y₁ [*b₁* = 0.43, *SE* = 0.03, 95% CI (0.37, 0.49)] than on Y₂ [*b₂* = 0.333, *SE* = 0.02, 95% CI (0.29, 0.38)]. The difference in slopes was significant [*Δ* = 0.10, *SE* = 0.02, 95% CI (0.05, 0.14), *F*(1, 528) = 16.90, *p* < 0.001, partial *η*^2^ = 0.03]. The results are shown at Table 6.

**Table 6:** Contrast test of positivity-ratio slopes for learning ability vs learning behavior (*n* = 531).

Statistic	Ratio → ability (Y₁)	Ratio → behavior (Y₂)	Δb	*SE*(Δ)	95% CI(Δ)	*F*	Partial *η^2^*
Slope (*b*)	0.43^***^	0.33^***^	0.10^***^	0.02	(0.05, 0.14)	16.90^***^	0.03
SE(*b*)	0.03	0.02					
95% CI(*b*)	(0.37, 0.49)	(0.29, 0.38)					

^***^Correlation is significant at the 0.001 level; Y₁ = learning ability self-efficacy; Y₂ = learning behavior self-efficacy. Δb = difference in unstandardized slopes (b₁ − b₂).

This pattern supported Hypothesis 3, indicating that the positivity ratio exerts a more substantial influence on ability-oriented efficacy judgments than on behavior-oriented efficacy.

The slope difference effect size (partial *η^2^* = 0.03) falls in the “small-to-medium” range (Cohen, 2013), but is educationally meaningful. Statistical significance does not necessarily imply practical importance. In real educational settings, this indicates that differences in emotional influence across ASE domains are noticeable but modest—enhancing positive affect may help students’ perceived learning ability somewhat more than behavioral control, yet the magnitude of this advantage remains relatively limited.

### 3.5 Graphical summary of the mediation results

**Figure 2:**
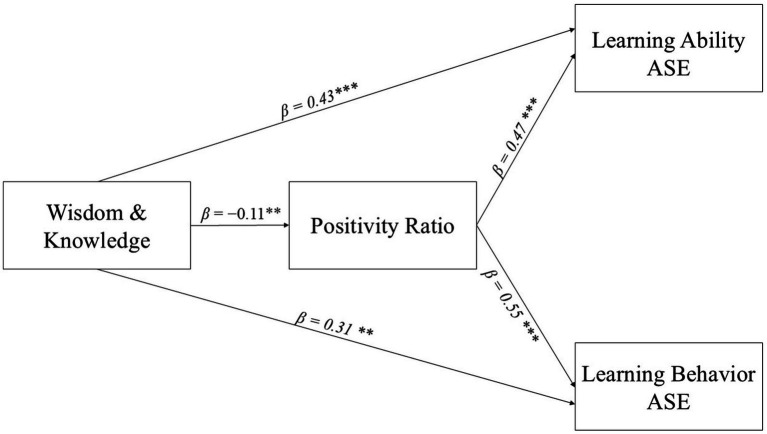
Correlation model between VIA-IS wisdom and knowledge, positivity ratio, and academic self-efficacy (*n* = 531); ^***^Correlation is significant at the 0.001 level. ^**^Correlation is significant at the 0.01 level (2-tailed).

**Figure 3:**
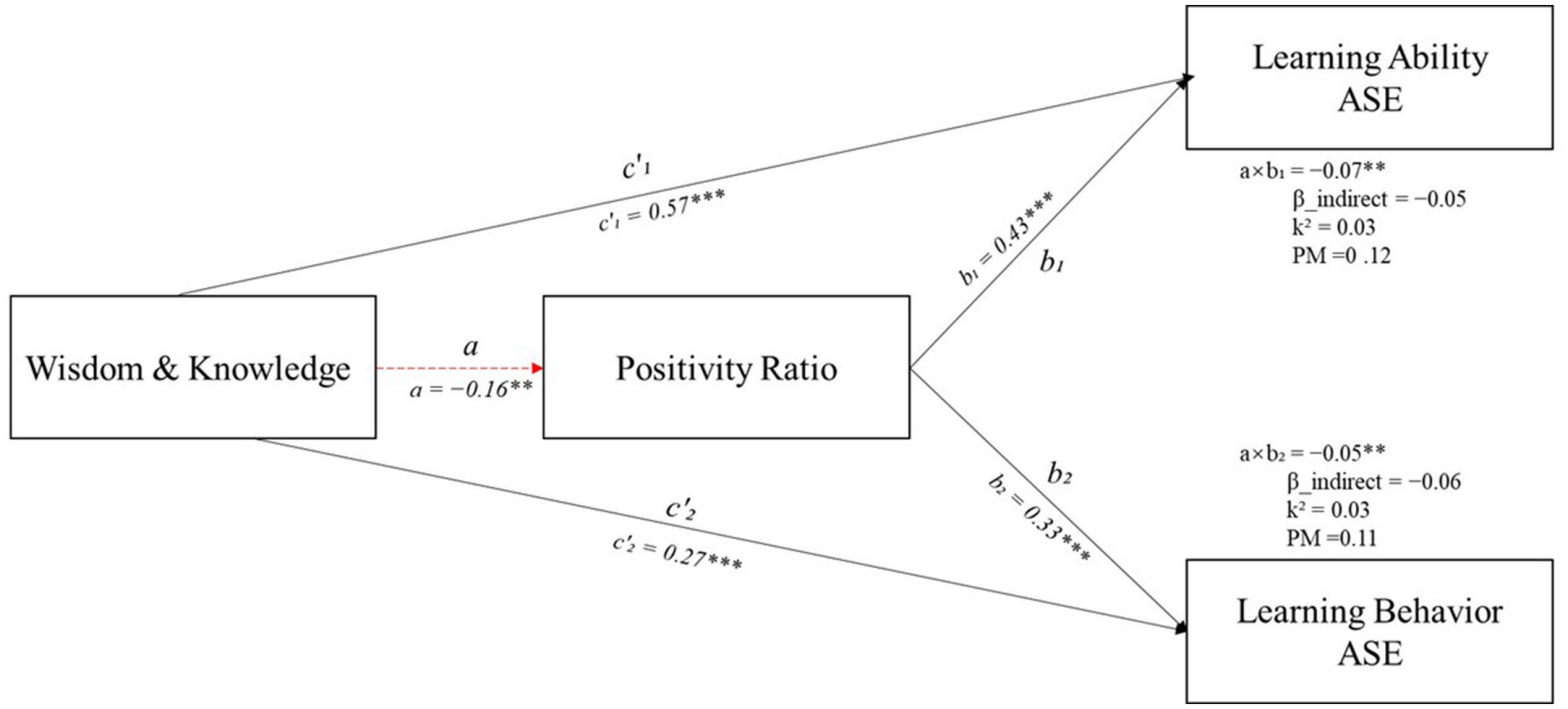
Path diagram of the mediation model showing the effects of wisdom and knowledge (X) on learning ability ASE (Y₁) and learning behavior ASE (Y₂) through the positivity ratio (M) (*N* = 531); ^***^Correlation is significant at the 0.001 level. ^**^Correlation is significant at the 0.01 level (2-tailed).

As shown in Figure 2, we specified a mediation model with VIA-IS Wisdom and Knowledge as the predictor (X), the positivity ratio as the mediator (M), and Learning Ability ASE (Y₁) and Learning Behavior ASE (Y₂) as the outcomes. The *a* path was negative (*β* = −0.11, *p* = 0.010), whereas both *b* paths were positive and significant (Y₁: *β* = 0.47, *p* < 0.001; Y₂: *β* = 0.55, *p* < 0.001). The corresponding unstandardized indirect effects were significantly adverse—Y₁: *a × b* = −0.07, 95% CI (−0.1130, −0.03); Y₂: *a × b* = −0.05, 95% CI (−0.09, −0.02)—indicating inconsistent (competitive) mediation. In addition, the GLM slope comparison showed a significant difference between the 2 *b* paths [Δ = 0.10, 95% CI (0.05, 0.14), *p* < 0.001], such that the effect of the positivity ratio on Y₁ exceeded that on Y₂. The mediation model path diagram is illustrated in Figure 3. As illustrated in Figure 3, the red dashed line signifies the negative mediating path (path a), while the black solid line denotes the positive main path and path b. The figure visually summarizes the key results reported in Sections 3.3 and 3.4: H2 was supported (both indirect effects were significant), and H3 was supported (the two *b* paths differed significantly in magnitude).

## 4 Discussion

The present study offers empirical evidence for an integrated strengths-emotion-efficacy framework in academic contexts. The findings indicate that wisdom and knowledge strengths exert both direct and indirect influences on students’ academic self-efficacy through emotional balance. Specifically, positive affect was found to partially mediate the link between cognitive strengths and efficacy beliefs, suggesting that emotional resources act as a bridge that amplifies the motivational benefits of intellectual strengths. Furthermore, the mediation effect was found to be more pronounced for learning ability efficacy than for learning behavior efficacy. This finding suggests that confidence in mastering knowledge is more immediately influenced by positive affective experiences. Conversely, the implementation of behavioral control may necessitate the provision of sustained strategic and contextual support. Collectively, these findings indicate a dual mechanism through which character strengths enhance academic confidence, operating through both cognitive resource activation and affective regulation. This dynamic interplay aligns with social cognitive and broaden-and-build theories, illustrating how personal strengths and emotions jointly sustain adaptive learning. Theoretically, the study contributes to clarifying differentiated pathways within academic self-efficacy; practically, it underscores the importance of integrating strengths cultivation and emotional training to foster holistic student development in higher education.

### 4.1 Summary of findings

This study examined the mechanism linking VIA-IS Wisdom and Knowledge → positivity ratio → academic self-efficacy (ASE) in a sample of Chinese university students, distinguishing between two ASE dimensions: Learning Ability ASE (Y₁) and Learning Behavior ASE (Y₂). All instruments demonstrated sound measurement properties: the TSES clearly separates ability and behavior factors (Liang, 2000; Zhen et al., 2017), and the revised Chinese PANAS has demonstrated reliability and validity in Chinese university samples (Crawford and Henry, 2004; Guo and Gan, 2010).

The main findings were as follows. First (H1), both Wisdom and Knowledge strengths and the positivity ratio were positively associated with both ASE dimensions (*p* < 0.01), consistent with social cognitive theory and control–value theory, which emphasize the role of emotional and cognitive resources in shaping efficacy beliefs and academic outcomes (Bandura, 1997; Pekrun et al., 2002; Schunk and DiBenedetto, 2020; Zimmerman, 2000). Second (H2), the positivity ratio mediated the link between Wisdom and Knowledge and ASE. However, this mediation was inconsistent or competitive: the *a* path (Wisdom and Knowledge → positivity ratio) was negative, while the *b* path (positivity ratio → ASE) was positive, producing an indirect effect opposite in sign to the direct effect (Zhao et al., 2010). Third (H3), multivariate GLM slope comparisons revealed that the effect of positivity ratio on Y₁ was significantly more potent than its effect on Y₂ [Δ = 0.10, *SE* = 0.02, 95% CI (0.05, 0.14), *F*(1, 528) = 16.90, *p* < 0.001]. This finding is consistent with the broaden-and-build theory, which posits that positive emotions yield immediate cognitive benefits (attention, flexibility, working, memory) that more directly map onto ability-related efficacy judgments (Ashby and Isen, 1999; Pintrich, 2000; Fredrickson, 2001).

Overall, the findings highlight a dynamic framework in which cognitive strengths and emotional balance jointly contribute to efficacy judgments, with evidence of both reinforcement and partial offset through competing mediation. From an effect-size perspective, the associations of emotional balance with ASE dimensions reached levels that are “educationally noticeable” (Cohen, 2013), while the competitive mediation highlights that strengths–emotion–efficacy are not a simple additive chain: cognitive strengths directly enhance ASE but may simultaneously reduce positivity under high standards, yielding a net effect that is partly offset.

### 4.2 Discussion of findings

(1) Direct effects (H1). The study found that Wisdom and Knowledge strengths (creativity, curiosity, judgment/critical thinking, love of learning, and perspective) were significantly and positively associated with both dimensions of ASE. Similarly, the positivity ratio (PA/NA) also showed significant positive correlations with both efficacy dimensions. These findings align with social cognitive theory, which identifies emotional and physiological cues as important sources of efficacy beliefs (Bandura, 1997; Schunk and DiBenedetto, 2020), and with control–value theory, which emphasizes the interplay between academic emotions and self-beliefs (Pekrun et al., 2002). In terms of the “strengths–learning” link, the results are consistent with prior evidence that character strengths in school contexts are positively related to classroom behavior, enjoyment of learning, and academic achievement (Wagner and Ruch, 2015). Moreover, strength-based interventions have shown potential to enhance motivation and adjustment in both primary/secondary and higher education settings (Lavy, 2020; Quinlan et al., 2012). The robust association between ASE and learning outcomes observed here also converges with meta-analytic evidence (Honicke and Broadbent, 2016).
(2) Mediation effects (H2). The findings confirmed the presence of a “strengths → emotions → efficacy” pathway but in the form of an inconsistent/competitive mediation: the path from Wisdom and Knowledge to positivity ratio was negative (a < 0), while the path from positivity ratio to ASE was positive (b > 0), resulting in a, b < 0 (Zhao et al., 2010). Whereas prior work has often reported uniformly positive mediation via affect (Fredrickson and Joiner, 2002), the competitive pattern may reflect high standards and evaluative pressure in authentic academic ecologies. A plausible explanation is that, in high-performance or high-evaluation academic contexts, individuals with strong cognitive strengths may engage in stricter self-evaluation and critical processing. This could temporarily heighten sensitivity to shortcomings and risks, thereby lowering emotional balance (a < 0), while the positive influence of emotions on efficacy judgments remains (b > 0). Such a structure of “direct enhancement plus partial offset via emotions” is consistent with conservation of resources theory, which posits that resource gains and losses can co-occur and are moderated by situational demands (Hobfoll, 2001). Compared to prior studies reporting predominantly positive mediation (e.g., positive emotions enhancing engagement and self-beliefs) (Fredrickson and Joiner, 2002), the present findings suggest that in authentic academic ecologies, “strength–emotion mismatches” or short-term emotional costs under high standards may arise, offering an explanation for variability in effect sizes across studies.

However, it is imperative to exercise caution when interpreting ratio-based affect indicators. Brown et al. (2013) critically reanalyzed the mathematical basis of the positivity ratio, demonstrating that the proposed “critical threshold” lacked empirical validity. Ratio scores are highly sensitive to small denominators and can inflate variance when negative affect values are low, raising concerns about statistical stability. Furthermore, affective balance is not inherently bipolar; positive and negative affect frequently function as partially independent systems (Diener and Emmons, 1984; Watson and Tellegen, 1985).

To address these limitations, a robustness check using the PA–NA difference score was conducted, yielding nearly identical mediation patterns. This finding substantiates the hypothesis that the observed effects are not merely artifacts of ratio scaling, but rather, they are indicative of a genuine affective pathway that links cognitive strengths and efficacy. Consequently, while the positivity ratio persists as a pragmatic heuristic, its interpretation ought to be judicious and substantiated by alternative indicators.

(3) Differential effects (H3). The slope comparison indicated that the effect of positivity ratio on ability-related judgments (Y₁) was more substantial than its effect on behavior/executive judgments (Y₂). This aligns with emotion–cognition theories: the immediate cognitive gains conferred by positive emotions (broadened attention, working memory, and cognitive flexibility) map more directly onto judgments of capability (“can I learn well?”), whereas Y₂ depends more on strategy use, time management, and self-monitoring processes, which are less sensitive to short-term emotional states and typically require longer cycles and contextual support to consolidate (Isen, 1993; Ashby and Isen, 1999; Pekrun et al., 2002; Schunk and DiBenedetto, 2020). Given that both dependent variables shared the same scale range (1–5, 11 items each), this pattern of “Y₁ more sensitive, Y₂ more delayed” has practical significance: in short-term interventions or high-pressure phases, enhancing positivity may first translate into gains in ability-related efficacy, whereas improvements in behavioral control are more likely to rely on strategy training and organizational support.
(4) Integration. Taken together, the findings suggest a dynamic framework for the “strengths–emotion–efficacy” pathway: Wisdom and Knowledge provide cognitive resources and are amplified by positive emotions to enhance ability ASE, but under performance and career pressures, strengths may also incur emotional costs, creating a structure of “direct enhancement plus partial offset through emotions.” This underscores the importance of advancing strength cultivation in parallel with emotion regulation and contextual design.

Regarding inconsistent/competitive mediation, one contextual explanation involves “short-term costs of high standards.” Learners with strong cognitive strengths may apply strict self-calibration and critical monitoring strategies (e.g., frequent comparison with high benchmarks, heightened error salience), which can temporarily increase tension and negative arousal, lowering PA/NA. Over longer timeframes, however, such critical processing may yield better strategies and mastery experiences, ultimately enhancing ASE. This aligns with COR theory’s emphasis on simultaneous resource gains and losses moderated by context (Hobfoll, 2001). Meanwhile, the evidence that “ability-related efficacy is more sensitive, behavior-related efficacy more delayed” fits with the idea that positive emotions first benefit attentional breadth and working memory (Isen, 1993; Ashby and Isen, 1999), while behavioral efficacy depends more on sustained strategy and time-management training. This suggests that intervention sequencing may prioritize “emotion → ability ASE” pathways initially, followed by “strategy training → behavior ASE” to produce cumulative gains.

### 4.3 Theoretical implications

The present study makes three theoretical contributions. First, it shows that character strengths contribute to ASE not only directly but also indirectly via positive emotions, extending broaden-and-build theory to academic contexts (Fredrickson, 2001; Fredrickson and Joiner, 2002; Wagner and Ruch, 2015). Second, the more substantial effect of the positivity ratio on Y₁ than on Y₂ supports the notion of differential sensitivity, whereby positive emotions exert more immediate influence on ability-related judgments, while behavior-related efficacy depends on longer-term regulatory processes (Isen, 1993; Ashby and Isen, 1999; Schunk and DiBenedetto, 2020). Third, by integrating strengths, emotional balance, and efficacy into a unified model, this study links conservation of resources theory (Hobfoll, 2001) with control–value theory (Pekrun et al., 2002), demonstrating how academic adaptation emerges through multi-level couplings of cognitive, emotional, and motivational resources. The finding of competitive mediation further suggests that contextual stressors and performance demands may modulate these relationships, helping explain inconsistencies across prior studies (Zhao et al., 2010).

From a cross-cultural perspective, these findings also underscore the contextual boundaries of generalization. Given that the sample was drawn from Chinese university students, whose educational environment is characterized by collectivist norms, competitive evaluation, and strong emphasis on academic excellence, the observed pattern of “direct enhancement but partial emotional offset” may partly reflect culturally embedded achievement pressure. In educational systems that prioritize autonomy, self-expression, and mastery goals (as seen in many Western contexts), positive affect may play a more uniformly facilitative role in efficacy formation. Future comparative studies could test whether the competitive mediation structure replicates across cultures or whether emotional balance mediates strengths–efficacy links differently under varying achievement ideologies.

At the theoretical level, the contributions can be summarized as “three couplings and two types of effects”: (a) Coupling 1: strengths (VIA-IS) and emotions (PA/NA) jointly predict ASE; (b) Coupling 2: the differential sensitivity of ability versus behavior ASE; (c) Coupling 3: coexistence of direct effects and competitive mediation. This layered framework integrates social cognitive theory (Bandura, 1997), broaden-and-build theory (Fredrickson, 2001), and control–value theory (Pekrun et al., 2002) within a single sample, while offering a culturally grounded interpretation of how emotional-cognitive couplings may vary across learning systems.

### 4.4 Practical implications

The findings carry several implications for educational practice. First, strength-based approaches: curricula and counseling should systematically identify and cultivate Wisdom and Knowledge strengths (e.g., inquiry-based learning, critical thinking exercises, interdisciplinary reading), thereby enhancing core cognitive resources (Quinlan et al., 2012; Wagner and Ruch, 2015; Lavy, 2020). Beyond the assessment phase, institutions of higher education have the opportunity to implement a structured “strengths training” program. This program aims to assist students in recognizing, applying, and refining their distinctive cognitive strengths through activities such as reflective journaling, peer feedback, and real-world problem-solving exercises. Such interventions have been demonstrated to enhance self-awareness and intrinsic motivation, thereby converting personal strengths into observable academic efficacy.

Second, emotional interventions: evidence-based practices such as mindfulness, loving-kindness meditation, positive writing, and cognitive reappraisal can reduce rumination and enhance positivity ratios, especially under high evaluative pressure, thus improving the translation of strengths into efficacy (Fredrickson et al., 2008; Sin and Lyubomirsky, 2009; Cohn and Fredrickson, 2010). The implementation of a university-based “emotion regulation workshop” as a short-term module within orientation or counseling programs has the potential to impart mindfulness, gratitude journaling, and reappraisal strategies. Empirical evidence demonstrates that such practices can function as a stress buffer, promote positive affect, and enhance learning confidence in challenging environments.

Third, belief construction: structured feedback, differentiated goals, and strategy training (including time management, self-monitoring, and reflection) can foster upward spirals of “belief–emotion–engagement” (Bandura, 1997; Komarraju and Nadler, 2013; Schunk and DiBenedetto, 2020). Fourth, contextual design: during exam or competition periods, institutional support for emotion regulation and time management may prevent mismatches between high strengths and high pressure that could otherwise reduce positivity ratios and weaken overall effects. Teachers can also adjust evaluative practices to emphasize formative feedback, thereby reducing threat appraisals and sustaining efficacy expectations (Pekrun et al., 2002).

Based on effect sizes and differential sensitivity evidence, a “three-phase” intervention cycle is suggested for universities: Phase 1 (2–4 weeks) focuses on positive emotion training (mindfulness, loving-kindness meditation, positive writing, reappraisal) to rapidly boost ability ASE; Phase 2 (4–8 weeks) incorporates learning strategy and time-management training to strengthen behavior ASE; Phase 3 (ongoing across semester) uses formative assessment and timely feedback to consolidate an upward spiral of belief–emotion–engagement (Sin and Lyubomirsky, 2009; Cohn and Fredrickson, 2010; Komarraju and Nadler, 2013). At the curriculum level, VIA-IS strengths identification can be integrated into inquiry-based learning and critical thinking modules (Wagner and Ruch, 2015; Lavy, 2020). During high-stakes exam periods, universities should also provide institutional support for emotion regulation and time management to prevent “strength–stress mismatches.” The proposed phased model constitutes a viable “strengths + emotion regulation” intervention framework, which universities can adapt within counseling, academic advising, or general education programs.

However, the practical implementation of this approach may encounter several challenges. Firstly, the temporal constraints and substantial academic obligations that students face could impede their ability to engage in multi-week interventions over an extended period. Secondly, faculty training and resource allocation are imperative. Teachers may require professional development to integrate their strengths and emotion-based components into regular teaching. Thirdly, the prevailing institutional culture may prioritize performance outcomes over emotional wellbeing, thereby reducing administrative motivation to adopt such programs. Addressing these barriers will require pilot testing, cross-department collaboration, and longitudinal monitoring to ensure both feasibility and long-term impact.

### 4.5 Limitations and future directions

Several limitations should be acknowledged.

First, the cross-sectional and self-report design limits causal inference and raises concerns about common method bias. The implementation of an online survey platform has the potential to exacerbate existing biases, both in terms of self-report and sampling. Despite the implementation of attention-check procedures, future research should utilize multi-wave or multi-source data collection methods, such as teacher ratings, classroom observations, and behavioral logs, to minimize common-method variance and enhance the validity of the research (Chemers et al., 2001; Oriol-Granado et al., 2017). Although Harman’s single-factor test suggested that common-method variance did not dominate the findings (Podsakoff et al., 2003), future research should adopt multi-wave, multi-source, or mixed-method designs to minimize potential method bias further and to strengthen the robustness of inferences.

Second, the study operationalized positivity as a PA/NA ratio. The “critical ratio” concept has been challenged (Brown et al., 2013), and ratio indices are sensitive to small denominators. In light of the persistent critiques regarding the mathematical instability of ratio metrics, future studies should employ cross-validation with alternative indicators, such as residual zed affect balance, and advanced modeling techniques, including polynomial regression or EMA designs. This approach will facilitate the testing of non-linear affect–efficacy relations. Collective integration of multiple affect indices with longitudinal and latent-variable frameworks would enhance the robustness and generalizability of future findings.

Third, contextual moderators warrant examination. Variables such as stress type (challenge vs. hindrance), social support, and disciplinary context may influence the strength and direction of “strengths–emotion–efficacy” pathways, as may the temporal sequencing of reverse effects (Pekrun et al., 2002; Schunk and DiBenedetto, 2020). Furthermore, the regression models utilized remain at a preliminary stage and do not account for measurement error or latent constructs. In the future, studies may benefit from the adoption of structural equation modeling (SEM) or multilevel modeling. These methodologies have the potential to enhance the capture of latent dynamics and contextual heterogeneity (Field, 2024).

Finally, the use of convenience sampling from Chinese universities limits generalizability. The gender composition of the sample (73% female) also suggests potential bias in ASE estimation and emotional reactivity patterns. Broader cross-institutional and cross-cultural samples are needed to assess model universality and boundary conditions (Pascoe et al., 2020). Intervention studies should also compare the effectiveness and cost–benefit of different combinations of strength-building, emotional training, and strategy guidance. Given that the theoretical contributions are rooted in the Chinese educational context, future research should explore whether similar patterns hold across individualistic and collectivistic cultures. This would allow for a test of the global applicability of the “competitive mediation” structure.

## Data Availability

The raw data supporting the conclusions of this article will be made available by the authors, without undue reservation.
