# Supplementary material for: From wisdom to efficacy: the mediating role of positivity ratio in the relationship between university students’ knowledge strengths and academic self-efficacy
Source: Front Psychol. 2025 Nov 25;16:1705454. doi: 10.3389/fpsyg.2025.1705454 (PMC12685654; doi:10.3389/fpsyg.2025.1705454)
Supplement: Supplementary file 1 [file Table_1.DOCX]

Supplementary Material

# Wisdom and Knowledge Subscale of the *Values in Action Inventory of Strengths (VIA-IS)*

Below are some thoughts and behaviors that students may have during their studies. Please **select the answer that best reflects your actual situation**. Each question has five response levels (1, 2, 3, 4, 5); you must choose only one answer per question and leave no question unanswered. There are no right or wrong answers—your true response is the best answer.(1 = "Strongly Disagree"; 2 = "Disagree";3 = "Neutral"; 4 = "Agree";5 = "Strongly Agree")

| Creativity | When others tell me how to do something, I unconsciously think of other ways |
| --- | --- |
|  | I don’t have any special motivation to do original work. |
| Curiosity | I  never feel bored. |
|  | I have very few interests. |
| Open-Mindedness | I only make decisions when I understand all the circumstances. |
|  | If I like a choice, I won’t consider other possibilities anymore. |
| Love of Learning | I do my best to participate in learning activities. |
|  | I often feel bored when reading non-fiction. |
| Perspective | People think I am smarter than my peers. |
|  | Others rarely seek my advice. |

# *Revised Positive and Negative Affect Schedule (PANAS)* to assess positive and negative affect, and calculate the Positive Ratio by Guo, M., & Gan, Y. Q. (2010)

Below are adjectives describing emotional states. Please reflect on your **current true emotional feelings**and select the level that matches the intensity of each emotion for you: (1 = "Very slight or none at all"; 2 = "A little"; 3 = "Moderate level"; 4 = "Quite a lot"; 5 = "Extremely intense")

| **Category** | **Specific Affects** |
| --- | --- |
| Positive Affect | Active |
|  | Enthusiastic |
|  | Happy |
|  | Elated |
|  | Excited |
|  | Proud |
|  | Joyful |
|  | Energetic |
|  | Attentive |
| Negative Affect | Ashamed |
|  | Sad |
|  | Afraid |
|  | Nervous |
|  | Scared |
|  | Guilty, |
|  | Irritable |
|  | Jittery |
|  | Annoyed |

# Academic Self-Efficacy Scale (TSES) developed by Liang Yusong (2000)

Below are some thoughts and behaviors that students may have during their studies. Please **select the answer that best reflects your actual situation**. Each question has five response levels (1, 2, 3, 4, 5); you must choose only one answer per question and leave no question unanswered. There are no right or wrong answers—your true response is the best answer. (1 = "Strongly Disagree"; 2 = "Disagree";3 = "Neutral"; 4 = "Agree";5 = "Strongly Agree")

| **ASE Category** | **Specific Items** |
| --- | --- |
| Learning Ability Academic Self-efficacy | I believe I can achieve high academic performance. |
|  | I believe I can achieve high academic performance. |
|  | I believe I can achieve high academic performance. |
|  | I can master classroom content promptly. |
|  | I can apply learned knowledge to real-world situations. |
|  | My understanding of my major is more comprehensive than peers. |
|  | I prefer challenging academic tasks. |
|  | I can effectively comprehend textbook and lecture materials. |
|  | I often choose difficult tasks that enhance knowledge, even if they require extra effort. |
|  | After poor exam performance, I calmly analyze my mistakes. |
|  | My self-perceived academic ability remains stable regardless of grades. |
| Learning Behavior Academic Self-efficacy | I test my knowledge by self-questioning during study sessions. |
|  | I connect new information with prior knowledge when problem-solving. |
|  | I frequently read without retaining meaning. |
|  | I integrate new knowledge with existing understanding while reading. |
|  | I struggle to stay focused during lectures. |
|  | I have difficulty summarizing key points from readings. |
|  | I highlight critical information to aid learning. |
|  | I synthesize knowledge across topics during exam preparation. |
|  | I try to record every word of lectures, even if irrelevant. |
|  | I recall lecture content to complete homework accurately. |
|  | I voluntarily practice end-of-chapter exercises to assess mastery. |
